# Barriers to rural households’ participation in low-skilled off-farm labor markets: theory and empirical results from northern Ethiopia

**DOI:** 10.1186/2193-1801-2-97

**Published:** 2013-03-09

**Authors:** Bharat P Bhatta, Torbjørn Årethun

**Affiliations:** Sogn og Fjordane University College, P.O. Box 133, Sogndal, NO-6851 Norway

**Keywords:** Rural households, Low-skilled off-farm labor market participation, Northern Ethiopia, Heckman’s two stage model, Entry barriers, Household characteristics, D10, J21, R20

## Abstract

Promotion of low-skilled off-farm rural labor market participation can be an important strategy to improve livelihoods and food security of the poor in developing countries. This paper investigates rural farm households’ participation in low-skilled off-farm labor markets with disaggregate data from a survey of 400 households in Tigray, the northern highlands of Ethiopia. Adopting Heckman’s two stage approach, we examined households’ decisions to participate or not in markets by probit model in the first stage and level of participation by ordinary least squares procedures in the second stage. The results show that households’ decision to enter into a labor market significantly depends on the characteristics of the households such as sex, age of the household heads and labor endowments in the households. Similarly, the level of participation in labor markets measured by the amount of off-farm wage income depends on labor endowments in the households and the place where the households are located. Since cash constrained rural households do not find themselves advantageous to participate in off-farm labor markets, the reduction of cash constraint is the major policy implication of the paper. This holds true in general for all cash constrained rural households in developing countries. Similarly, the empirical results in the paper suggest removal of locational barriers to access labor markets. This helps them to earn off-farm income. It is necessary to eliminate (or at least reduce) obstacles for rural households to enter into a market of off-farm wage earning activities. This holds true in general for all rural households in developing countries. This paper is therefore expected to contribute to frame appropriate policy that promotes participation in low-skilled off-farm rural labor markets in developing countries where many rural households are not only poor but also low-skilled.

## Introduction

Labor is one of the most important assets of a rural household. In many cases, it is the only productive asset for land-poor households available for allocation in developing countries. Decision of the households to allocate their labor is therefore important for their livelihood. Because poor people in a developing country like Ethiopia face continuously declining agricultural productivity due to excessive dependence on agriculture and environmental factors, it is equally important whether the households allocate their labor to off-farm labor markets or not. There are a number of studies that report a considerable and rising share of off-farm income in total household income (Hageblade et al., 2007; de Janvry and Sadoulet, 2001; Ruben and van den Berg, 2001). In addition, the importance of off-farm income has been extensively documented in literature, for example, off-farm income and employment in rural Honduras (Isgut, 2004), off-farm employment and poverty in rural El Salvador (Lanjouw, 2001), off-farm labor allocation and decisions in small scale rural households in Zimbabwe (Matshe and Young, 2004), poverty and rural off-farm economy in Oromia, Ethiopia (van de Berg and Kumbi, 2006), rural off-farm employment and income diversification in Columbia (Deininger and Olinto, 2001) and so on. As a result, promotion of off-farm rural labor market participation can be the important strategy to improve livelihoods and food security of the poor in developing countries in general and Ethiopia in particular. However, the share of rural households reporting specific skills such as carpentry and sewing was about 3% in the study area, indicating the importance of low-skilled off-farm labor markets, but not an off-farm labor market in general. Similarly, the share of rural households reporting income from off-farm labor market participation was only 54% in the study area implying that there were nearly 50% households which did not participate in off-farm rural labor markets. The study is significantly important in this context in order to explore the barriers for the rural households to enter into low-skilled off-farm wage earning activities. Participation of rural households on off-farm labor markets varies significantly across/within countries. The same is true with the factors influencing the participation. However, there are hardly any studies that focus on low-skilled labor markets.

In addition to the studies in this line of inquiry cited above, there are many other studies with emphasis on investigating the entry barriers to off-farm rural labor markets in developing countries, for example, determinants of rural labor market participation in Tanzania (Mduma and Wobst, 2005), determinants of off-farm earning (Abdulai and Delgado, 1999; Escobal, 2001), income diversification and entry barriers in the Tigray region of northern Ethiopia (Woldenhanna and Oskam, 2001), factors determining off-farm activities and income of rural households with focus on education in Mexico (Yunez-Naude and Taylor, 2001), patterns, determinants and impacts of income diversification in rural Nigeria (Babatunde and Qaim, 2009), and so on. However, none of these studies clearly focus on low-skilled off-farm labor markets whereas the majority of rural households do not have any specific skills as in Tigray. The study in this paper focus on low-skilled off-farm labor markets and addresses the problem from a theoretical and behavioral perspective. The study uses a behavioral model where a household maximizes utility subject to constraints due to time, cash, production and budget which is a reasonable assumption for rural households in developing countries. This study also empirically addresses the problem using a model which is consistent with rural households’ behavior in participating off-farm wage earning activities in developing countries. In this respect, the study is significantly different from other studies.

The objective of the paper is to examine rural households’ participation in low-skilled off-farm labor markets and to explore the factors that influence (or are correlated to) the participation. Following Heckman’s approach (Heckman, 1979; see also Wooldridge, 2002), this paper adopts a two-stage procedure. In the first stage, rural farm households’ decision to enter into a labor market and factors influencing these decisions are examined by a probit model. In the second stage, their level of participation and the factors that influence the level of participation are investigated by the ordinary least squares (OLS) method. Abdulai and Delgado (1999) have used the similar approach to investigate determinants of off-farm earnings in northern Ghana. de Janvry and Sadoulet (2001) have used a multinomial model to analyze the role of off-farm activities in Mexico. The studies by Isgut (2004), Yunez-Naude and Taylor (2001), Lanjouw (2001), and Ruben and van den Berg (2001) have used probit and logit models to examine rural households’ participation in various off-farm activities. We investigate the problem based on a survey of 400 households carried out in Tigray in 2003. The paper also highlights a theoretical contribution that cash constrained rural households face shadow wage rate within the households which are higher than the market wage rate making them not beneficial to participate in a labor market. In addition, the results in the paper contribute to empirical literature because no related recent evidence is available for Tigray using the same approach. It is necessary to eliminate (or at least reduce) obstacles for rural households to enter into a market of low-skilled off-farm wage earning activities. This holds true in general irrespective of locations of households.

The remainder of the paper is organized as follows. Section 2 outlines the theoretical and empirical bases underlying the study. Section 3 describes the data and estimations. Section 4 presents the empirical results and discussions. Finally, Section 5 concludes the study with some policy implications.

## Theoretical framework and the model

As households simultaneously take decisions regarding investment, production, consumption, and labor supply, a household perspective is the most appropriate to investigate factors influencing rural households’ low-skilled off-farm labor market participation. Since a typical household in resource poor rural economies normally faces time, production, cash, and budget constraints, the household model must take those constraints into account (cf. Sadoulet and de Janvry, 1995). Our study area, Tigray, is not an exception. We therefore assume that a household maximizes utility, which is a function of consumption of agricultural goods, market-purchased commodities and leisure, subject to the above mentioned constrains:

where U is a quasi-concave, continuous and non-decreasing utility function, X_a_ represents a vector of agricultural goods, X_m_ is a vector of commodities purchased from markets, and X_l_ is minimum time necessary for household and social activities, leisure and so on. The household maximizes utility subject to the following constraints:

1. Production constraint:
  where Q is the quantity of agricultural commodities produced by the household, L is total amount of time spent on its own farm, X_f_ represents households’ use of market-purchased agricultural inputs such as fertilizer, and  is the amount of fixed assets such as land.
2. Time constraint: T = F + X_1_
  where T is total time of the household, F is time available for work after allocating the minimum necessary time for household and social activities, leisure and so on. So F-L is the amount of time that a household can sell in labor markets.
3. Cash constraint:
  where *P*_*f*_ is price of market-purchased inputs such as fertilizer, w denote the market wage rate, and  is total cash (in fixed amount) available for the household from its own savings, remittances, loan from money lenders, friends and relatives and so on. Here we assume that there is no market for formal credits. The household needs cash to buy inputs such as fertilizer from markets so it faces cash constraint at the start of the season.
4. Budget constraint:
  where *P*_*m*_ is price of market-purchased goods and *P*_*a*_ is price of agricultural commodities.

Here we assume a seasonal labor market. This is a reasonable assumption for the study area. In rainy and harvesting seasons, for example, everybody works in their own farms and laborers are hired in or out very rarely.

Combining the constraints (1), (2), and (4) to get a full budget constraint:

Setting Lagrange function to maximize household utility subject to the full budget constraint and credit constraint:

Differentiating the Lagrangian function with respect to labor (L) and setting the equation equal to zero gives the first order condition of household utility maximization as follows:

where MVP_L_ stands for marginal value product of labor in their farm. The above relation clearly shows that the household wage rate deviates from market wage rate if the household faces a binding cash constraint (i.e., μ>0). As a result, the household cannot get rid of shadow wage rate. As long as the cash constraint is binding (i.e., μ≠0), household shadow wage rate is higher than the market wage rate making the household not beneficial to participate in a labor market. It is the shadow wage rate which decides whether a household would participate or not in a labor market. If the shadow wage rate lies within “price band”(cf. Key et al., 2000), the household becomes self-sufficient and will not participate in a market (cf. Sadoulet and de Janvry, 1995). In contrast, the household will participate in a market if the shadow wage rate lies above the price band. The relation has an important policy implication that it is the cash constraint that obstructs rural households from entering into a market of off-farm wage activities. It is necessary to remove the cash constraint of rural households in developing countries so that the rural poor will participate in labor markets and become less dependent on subsistent farming resulting in less land degradation.

As can be seen above, labor market participation by a rural household depends on the width of the price band and the value of shadow wage rate. The width of the price band depends on transaction costs, shallow markets, price risk and risk aversion. Shadow wage rate is determined internally within the household and depends on household characteristics and household specific indicators of the market and resource endowments of the household. Hence the estimable econometric model for labor market participation by rural households can be written as:

where LMP stands for labor market participation by rural households, M the vector of factors related to characteristics of the market such as transactions costs and information asymmetries and, h^z^ the vector of household characteristics such as age, sex, education of household head and consumer worker ratio, R the vector of household income and resource endowments such as labor, oxen, livestock, and land owned, L the vector of village level factors and public goods to account for fixed and random effects, and U the vector of unobserved factors with expected value of zero. If any of the variables in those groups are significant, it indicates imperfections in the markets that the rural households face. Otherwise the markets function reasonably well.

A rural farm household first decides to enter or not into an off-farm labor market. Having entered into the market, the next question comes how much the household earns from the market. The OLS model is applicable if all the households participate in markets. All the households do not participate in off-farm labor markets because some households may not find themselves advantageous to participate (voluntary nonparticipants) (cf. theoretical model in Section 2) while others may not get the opportunity to participate due to market conditions (involuntary nonparticipants). The estimation of OLS regression excluding the non-participants from the analysis introduces a sample selection bias to the model. The questions addressed in the paper are therefore investigated by using Heckman’s two stage model (Heckman, 1979; see also Wooldridge, 2002). The first stage examines whether farm households decide or not to participate in markets and factors that affect them to come up with that decision using a probit model. The probit model estimates probabilities of a household to participate in markets. The second stage analyzes level of participation by OLS model. In the second stage, the dependent variable is continuous and inverse mills ratio (IMR) was also included as one of the explanatory variables in the model in order to check for selection bias. The decision and level of participation by the rural farm households are discussed separately below.

## Data and estimation

The study in this paper uses primary data from a sample of 400^a^ households in 16 *tabia*^b^ located in Tigray. Tigray is estimated to have a total area of 80,000 sq. km. Altitude ranges from 3900 meters in the Southern zone to 500 meters in the Eastern zone. Tigray consists of 4 administrative zones, 35 districts (*weredas*^c^), 1089 *tabias* and 74 towns. Mekelle is the capital city of Tigray. The altitude of the study areas ranges from 1750 to 2750 meters above sea level. The climate in Tigray is highly unpredictable characterized especially by unreliable rainfall. The Tigray region faces sparse, extremely erratic and highly uneven distribution of seasonal rainfall and frequent drought. Severe droughts causing famine have affected the region approximately every tenth year through this century (REST and NORAGRIC, 1995). The amount of rainfall increases with altitude and from east to west, and decreases from south to north. Average rainfall varies from about 200 mm per year in the northeast lowlands to over 1000 mm per year in the south western highlands. Rainfall starts in late June/early July and ends in late August or early September. Most of the rainfall occurs from June to September.

Samples were selected adopting a stratified random sampling approach based on market access, population density, rainfall, and irrigation projects. First 16 *tabias* were selected for a household survey using a stratified sampling method. Lists of all households were obtained from those *tabias* selected and simple random sampling approach was adopted to select 25 households from each *tabia*. The survey was carried out in 2003*.* Table 1 illustrates descriptive statistics of the variables.

**Table 1:** **Descriptive statistics of variables used in econometric analysis**

Variables	Type	Mean	Std. Dev.	Min.	Max.
Distance of household from *wereda* headquarters (time in minutes)	C	165.8	96.4	3	480
Good access to road =1, 0 = otherwise	D	-	-	0	1
Close to market =1, 0 = otherwise	D	-	-	0	1
Male =1, 0 =otherwise	D	-	-	0	6
Age of household head	C	53.8	14.4	24	98
Education of household head	D	-	-	0	6
Female labor in household	C	1.3	.7	0	5
Male labor in household	C	1.1	.9	0	4
Dependency ratio	C	2.3	1.1	1	7
No of oxen in a household	C	.9	.9	0	5
Tropical livestock unit in a household	C	2.6	2.8	0	15.9
Labor market participation, yes=1, 0 = otherwise	D	-	-	0	1
Own land holding	C	1.1	.7	.13	6.5
Southern, yes =1, 0 = otherwise	D	-	-	0	1
Eastern, yes =1, 0 = otherwise	D	-	-	0	1
Central, yes =1, 0 = otherwise	D	-	-	0	1
Western, yes =1, 0 = otherwise	D	-	-	0	1
Labor income in Birr	C	169.1	324.4	0	2400

Where C = continuous, D = dummy, -= not applicable.

## Results and discussion

Here we present the results and discuss them. A substantial body of empirical results was generated using an iterative model building approach. The paragraphs that follow summarize the results of the models having the best specification. Here, it should be noted that some of the models contain coefficient estimates of low level of statistical significance; the inclusion of such coefficients, all of them have the expected signs, did not affect the remaining estimates. We took misspecification problems such as non-normality of residuals, heteroscedasticity, multicollinearity, omitted variables, and wrong functional form into account while estimating (Gujarati, 2003; Wooldridge, 2002).

The Jarque-Bera normality test and chi-square test of goodness of fit indicated that the residuals were normally distributed*.* According to variance inflation factors (VIF), which all were less than 10, the models did not have the serious problem of multicollinearity.

### Decision to participate

The probit results explaining the household’s decision to participate in low-skilled off-farm labor markets are reported in Table 2.

**Table 2:** **Probit results of households’ decision to participate in labor markets**

Variable	Coefficeint	Std. err.	t-stat.
Distance to wereda headquarters	−6.30E-04	8.60E-04	−0.73297
Market access (good=1, 0=otherwise)	−0.19402	0.16333	−1.1879
Road access (good=1, 0=otherwise)	3.44E-02	0.152	0.22629
Sex of household head (male =1, 0=female)	0.4611	0.19552	2.3583***
Age of household head	−1.47E-02	5.15E-03	−2.8573***
Consumer worker ratio	4.58E-02	8.19E-02	0.5595
Education of household head	−0.26426	0.16636	−1.5885
Male labor in the household	0.17305	9.04E-02	1.9134*
Female labor in the household	0.24278	0.10314	2.3539***
No of oxen in the household	−6.00E-02	0.11458	−0.52382
Total livestock unit holding	2.89E-02	4.23E-02	0.6834
Land owned	0.11759	0.10883	1.0805
Population density	−0.20783	0.16592	−1.2526
Estern zone	2.89E-02	0.2193	0.13189
Central zone	0.19045	0.19859	0.95901
Southern zone	0.37269	0.2141	1.7407*
Constant	7.37E-03	0.47096	1.57E-02

*. p-value<10%.

***. p-value<1%.

In line with the expectations, the coefficients of male head of the household, male labor in the household, female labor in the household, and southern zone were significantly positive meaning that male headed households having more male and female labor which are located in southern zone significantly increased the likelihood of participation of the rural households in off-farm labor markets. The male headed-households were more likely to participate in a labor market than female-headed households. The reason could be that female-headed households often lack adult labor to participate and household head himself often participates in labor markets. Further, as we had expected, probability of participation increased with male and female labor endowment in households implying that households did have idle labor which can be supplied to earn extra income. Another reason could be that more labor force relative to land endowments of a household brings declining returns to labor in their own farm and thus making them more beneficial to participate in labor markets. Southern zone was also significantly positive implying that the likelihood of participation by households located in Southern region was higher compared to households located in Western region because of village level fixed and random effects. This result is in line with the finding of Abdulai and Delgado (1999), who used the same approach to investigate the similar problem in northern Ghana, that infrastructure and population density are significantly related with the probability of off-farm labor market participation. As expected, probability of participation significantly decreased with age of a household head because younger household heads are more likely to be able to work hard compared to older people. The reason for this could be that working as labor requires physical strength. The empirical results of the probit model in the first stage are consistent with the outcome of the theoretical model derived in Section 2 that a rural household’s entry into off-farm labor markets depends not only on market wage rate, but also perhaps more importantly, on household characteristics, income and the number of people in working age belonging to the household and location of the household.

### Level of participation

Table 3 presents the results of the regression model with the amount of off-farm wage income as a dependent variable. Male and female labor in the household, and zonal dummies had significantly positive relationship with the amount of income earned from labor markets. Hence, male and female labor force in the household and locational factors not only influence households to enter into labor markets, but also contributes to higher average non-farm wage income. In this stage, sex and age of the household were no longer significant but two other zonal dummies were significant. Sex of household head was no longer significant, although it was significant in deciding to participate in labor markets. It gives much sense emphasizing the role of household head to decide to participate in labor markets, while both the amount of wage income and the level of participation increased with the number of males and females in working age within the household. This is in line with the expectation for the reasons explained above in the probit model. Similarly, the coefficients of zonal dummies were significantly positive implying the households’ levels of participation in labor market were significantly higher in Eastern, Central, and Southern zone compared to that of Western region because of village level fixed and random effects.

**Table 3:** **OLS results of households’ level of participation in labor markets**

Variable	Coefficeint	Std. err.	t-stat.
Distance to wereda headquarters	8.59E-02	0.15522	0.55342
Market access (good=1, 0=otherwise)	53.746	36.854	1.4584
Road access (good=1, 0=otherwise)	35.387	29.433	1.2023
Sex of household head (male =1, 0=female)	23.857	26.669	0.89458
Age of household head	−0.99506	0.8828	−1.1272
Consumer worker ratio	−1.2641	12.514	−0.10101
Education of household head	−41.503	29.904	−1.3879
Male labor in the household	36.513	15.879	2.2995**
Female labor in the household	37.203	18.398	2.0221**
No of oxen in the household	−1.5195	21.666	−7.01E-02
Total livestock unit holding	5.2178	7.3931	0.70577
Land owned	−14.395	19.041	−0.75603
Population density	11.254	39.971	0.28154
Estern zone	170.08	45.196	3.7632***
Central zone	69.434	27.751	2.5021***
Southern zone	154.9	52.347	2.9591***
Lamda (IMR)	193.07	19.556	9.8724***
Constant	−23.942	72.203	−0.33159

**. p-value<5%.

***. p-value<1%.

Since the inverse mills ratio (IMR) was highly statistically significant, there was sample selection bias implying that an OLS model that uses only the households participating in the labor markets lead to biased results.

The statistical significance of the variables in the models indicates that the rural households in Tigray face imperfect markets. Market imperfections lead to non-separable production, consumption and labor supply decisions. A rural household’s participation in off-farm labor markets depends not only on market wage rate, but also, perhaps more importantly, on household characteristics, income and resource endowments of the household, and location of the household. Conclusions from the neoclassical model therefore do not hold in resource-poor rural economy such as Tigray. The imperfections in markets limit the optimum production and consumption choices.

## Summary and conclusions

Labor is one of the most important assets of rural households. In many cases, it is the only productive asset for land-poor households available for allocation in developing countries. Allocation of the labor in households is therefore important for their livelihood and food security. Because poor people in a developing country like Ethiopia face continuously declining farm productivity due to excessive dependence on agriculture and environmental factors, it is very important whether the households allocate their labor to off-farm labor markets or not. Additionally, since most of the households did not have any specific skills and had very low level of education (most of them are illiterate) in the study area, barriers of entering low-skilled off-farm labor market is important. The aim of the study in this paper was therefore to examine rural farm households’ participation in low-skilled off-farm labor markets and to investigate factors that influence (or is correlated to) the participation. First we theoretically investigated the problem by using a household model where a rural household maximizes utility subject to constraints due to time, production, cash and budget. The outcome of the household model indicated that the household wage rate deviates from the market wage rate if the household faces a binding cash constraint (i.e., μ>0). As a result, the household cannot get rid of shadow wage rate. As long as the cash constraint is binding (i.e., μ≠0), household shadow wage rate is higher than the market wage rate making the household not beneficial to participate in a labor market. It is the shadow wage rate which decides whether a household would participate or not in a labor market. If the shadow wage rate lies within “price band” (cf. Key et al., 2000), the household becomes self sufficient and will not participate in a market (cf. Sadoulet and de Janvry, 1995). In contrast, the household will participate in a market if the shadow wage rate lies above the price band. This relation has an important policy implication that it is the cash constraint that obstructs rural households in entering into a market of off-farm wage activities. It is necessary to remove the cash constraint of rural households in developing countries so that the rural poor will participate in labor markets and become less dependent on subsistent farming resulting in less land degradation. This holds true in general.

As discussed above, off-farm labor market participation by a rural household depends on the width of the price band and the value of shadow wage rate. The width of the price band depends on transaction costs, shallow markets, price risk and risk aversion. Shadow wage rate is determined internally at the household which depends on household characteristics and household specific indicators of the market and resource endowments of the household. Finally, it was shown that a rural household’s participation in off-farm labor markets depends not only on market wage rate, but also, perhaps more importantly, on household characteristics, income and resource endowments of the household, and location of the household. Conclusions from the neoclassical model therefore do not hold in resource-poor rural economy such as Tigray which are characterized by market imperfections. The imperfections in markets limit the optimum production and consumption choices. We used cross-sectional data of 400 households obtained from a household survey carried out in Tigray in 2003. We adopted Heckman’s two stage approach to investigate the problem empirically.

Factors related to human resource endowments such as sex and age of household head, male and female labor in household significantly influence farm households to participate in low-skilled labor markets. Participation in labor markets increases with male household head, male and female labor endowments in a household while decreases with age of a household head. Once the household enters labor market, only male and female labor endowments determine the level of participation. Similarly, locations where the households are situated also significantly affect the decision and level of participation in labor markets. It seems that households located in remote areas are less likely to participate in labor markets so policies towards integrating remote areas with urban areas through infrastructure development are highly desirable.

## Endnotes

^a^The entire analysis in this paper however considers only 372 households due to incompleteness of data and respondent dropouts.

^b^*Tabia* is the local name for a community. *Tabia,* village and community therefore are used interchangeably in this paper.

^c^*Wereda* is the local name for a district.
